# Study on the effect factors of discharge readiness of total hip arthroplasty patients

**DOI:** 10.3389/fmed.2024.1405375

**Published:** 2024-08-23

**Authors:** Pei Liu, Weiqiang Chen, Ying Shan, Liang Dai, Xianglan Qin, Haoze Yang, Xingchen Ji, Zhen Tan, Fei Yu

**Affiliations:** ^1^Department of Bone & Joint Surgery, Peking University Shenzhen Hospital, Shenzhen, China; ^2^National & Local Joint Engineering Research Center of Orthopedic Biomaterials, Shenzhen, China; ^3^Shenzhen Key Laboratory of Orthopedic Diseases and Biomaterials Research, Shenzhen, China; ^4^Clinical Research Academy, Peking University Shenzhen Hospital, Shenzhen, China

**Keywords:** total hip arthroplasty, discharge readiness, Harris score, self-care ability, ERAS concept

## Abstract

**Introduction:**

In order to explore the correlation between discharge readiness and Harris score or self-care ability of patients undergoing total hip arthroplasty (THA) based on the enhanced recovery after surgery (ERAS) concept. We carried out this single center retrospective study.

**Methods:**

We enrolled 331 patients who underwent THA. These patients were divided into the higher score group and the lower score group according to median discharge readiness score. After the baseline data of these patients were compared, the effect factors of discharge readiness of these patients was analyzed through univariate and multivariate logistic regression analyses and mixed effects models.

**Results:**

The results demonstrated that there was a correlation between discharge readiness and changes in Harris score 30 days after discharge (compared with that before surgery) in these patients. Besides, the Harris score and self-care ability 30 days after discharge were higher than those at the time of discharge. In addition, patients in the higher score group exhibited a higher Harris score compared with those in the lower score group. From the evaluation at different time points after discharge, there was a significant difference in the Harris score between both groups.

**Discussion:**

It can be inferred that the discharge readiness of patients undergoing THA was correlated with the Harris score but not with the self-care ability. These results are expected to provide guidance for the physical and mental recovery of patients undergoing total hip replacement under the ERAS concept. Furthermore, these findings may contribute to higher diagnosis, treatment, and nursing levels of orthopedic medical staff.

## 1 Introduction

Total hip arthroplasty (THA) is a surgical treatment method for resolving advanced hip joint diseases with obvious pain, severe functional impairment, and ineffective conservative treatment caused by various reasons (1). Patients can regain a well-functioning hip joint after THA, which has been considered one of the most successful surgical procedures in the 20th century (2). The concept of enhanced recovery after surgery (ERAS) was first proposed by Dr. Kehlet, a Danish doctor, in 1997 and has been popularized worldwide. The ERAS concept was proposed based on multidisciplinary and multimodal methods, ultimately achieving accelerated recovery reduced incidence of complications, and improved prognosis (3). The ERAS concept was initially applied to patients undergoing abdominal and colorectal surgery, but later extended to other specialties, such as orthopedic surgery, especially hip and knee joint arthroplasty (4). Preoperative education/physical therapy, rational selection of anesthesia technology, optimization of multimodal analgesia, prevention of urinary retention and incidence of catheterization, active management of risks related to blood loss and deep vein thrombosis, and early mobilization of patients are all intervention methods under the ERAS concept. These methods may affect the physical and mental recovery of patients undergoing hip and knee replacement (5). Therefore, the ERAS concept can be adopted in any perioperative period for patients undergoing hip replacement surgery.

The concept of discharge readiness was proposed earlier than the ERAS concept. It was initially proposed by Fenwick et al. (6) in 1979 to identify whether patients were ready for discharge from both medical and patient perspectives and comprehensively evaluate the physiological, psychological, and social indicators of patients upon discharge through medical staff. Further, it can be employed to analyze the support that families and society can provide to patients and make judgments on their ability to return to society for subsequent rehabilitation. In recent years, discharge readiness has also received increasing attention with the extensive application of the ERAS concept. Additionally, patients with high discharge readiness scores are more significantly correlated with early rehabilitation and better prognosis under the ERAS concept, thus contributing to the comprehensive research on these two concepts (7). Discharge readiness can be regarded as an assessment indicator of a patient’s state before discharge, and it can be utilized to identify factors that may affect discharge readiness, and, to some extent, affect the promotion of ERAS concepts in related procedures (8). The focus of most existing studies is placed on the readiness for discharge in the field of orthopedics. YaDeau et al. (9) maintained that general anesthesia may be related to the discharge readiness of patients after foot and ankle surgery. Compared with patients receiving spinal anesthesia, those receiving general anesthesia can be discharged earlier. Sztain et al. (10) found that for patients undergoing unicompartmental knee arthroplasty, compared with continuous femoral nerve block, continuous adductor canal block can improve the patient’s conformity with predetermined standards and discharge criteria. The Harris score is a relatively objective scoring standard for evaluating hip joint functions. It is often used to evaluate changes in hip joint functions before and after surgery. The higher the Harris score, the better the hip joint function (11). The self-care ability of patients is related to their quality of daily life. This indicator can be quantified by the Activities of Daily Living (ADL) score. The higher the ADL score, the stronger the self-care ability (12). However, there are few reports on the discharge readiness in patients undergoing THA, especially the correlation between discharge readiness and Harris score or self-care ability.

In this study, we enrolled 331 patients who underwent THA in Department of Bone & Joint Surgery at Peking University Shenzhen Hospital from January 2021 to February 2023. These patients were divided into the higher score group and the lower score group according to median discharge readiness score. In order to explore the correlation between discharge readiness and Harris score or self-care ability of these patients. These results are expected to provide guidance for the physical and mental recovery of patients undergoing THA under the ERAS concept. Furthermore, these findings may contribute to higher diagnosis, treatment, and nursing levels of orthopedic medical staff.

## 2 Materials and methods

### 2.1 Type of study

Single center retrospective study. A total of 331 patients who underwent THA in Department of Bone & Joint Surgery at Peking University Shenzhen Hospital from January 2021 to February 2023 were included in this study.

### 2.2 Grouping

After sorting based on their discharge readiness score, the median score was selected as the cutoff point. Based on that, these patients were divided into the lower score group (*n* = 189) and the higher score group (*n* = 142). This study was approved by the Ethics Committee of Peking University Shenzhen Hospital. The study complies with all regulations in clinical research.

### 2.3 Clinical resources

The baseline data were captured by the HIS system, including the discharge readiness score, age, gender (male or female), BMI, height, weight, education level (illiterate/primary school, technical secondary school/secondary school, and college/university/master’s degree), marital status (married, divorced/widowed), length of hospital stay, anesthesia method (general anesthesia, lumbar spinal cord/spinal canal anesthesia), adverse events within 30 days after discharge, comorbidities, self-care ability (at admission, discharge, and 30 days after discharge), Harris score (at admission, discharge, and 30 days after discharge), discharge satisfaction, rehabilitation exercise compliance (discharge and 30 days after discharge) of these patients were collected.

### 2.4 Inclusion and exclusion criteria

The inclusion criteria included: ① patients aged 18 ≤ age ≤ 85 years; ② patients receiving initial unilateral THA; ③ patients with the ability to understand and cooperate with relevant instructions and complete the evaluation; ④ patients without severe organ dysfunction (Organ dysfunction can lead to patient death or poor medical outcomes, and these individuals should prioritize saving lives rather than undergoing total hip arthroplasty to improve their quality of life); ⑤ patients with the ASA grade ≤ 3; ⑥ patients with complete follow-up data within 30 days after discharge; and ⑦informed consent was obtained from the patient(s) for the PUBLICATION of clinical data and other data. The exclusion criteria included: ① patients with cognitive disorders, such as Alzheimer’s disease, which may hinder their participation and evaluation; and ② patients with other illnesses that may require treatment during hospitalization.

### 2.5 Collection of main research data

The discharge readiness score was obtained using the Readiness for Hospital Discharge Scale (RHDS). The Activities of Daily Living (ADL) score can be rated as 0–100 points, with higher scores indicating stronger ADL abilities (It is equal to the changing of Barthel index). The Harris score was obtained using the Harris Hip Rehabilitation Scale. The rehabilitation compliance of these patients was evaluated based on the previous research methods of our research group (13).

### 2.6 Statistical methods and data analysis

SPSS 18.0 was used for data analysis. The metrological data that were normally distributed were represented by mean ± standard deviation, and the differences between the two groups were compared using the two-sample *t*-test. In contrast, the metrological data that were not normally distributed were represented by the median (interquartile interval), and the differences between the two groups were compared using the Mann–Whitney U test. The counting data were expressed in frequency (percentage), and intergroup differences were analyzed through the χ2 test. The correlation between discharge readiness and Harris score or self-care ability was analyzed by univariate logistic regression and multivariate logistic regression analyses, respectively. In the multivariate logistic analysis, the adjusted covariates included age, gender, height, weight, marital status, length of hospital stay, anesthesia methods, comorbidities, and postoperative rehabilitation exercise compliance. The correlation between discharge readiness and Harris score or self-care ability over time was analyzed based on a mixed effects model. The adjusted covariates included age, gender, height, weight, marital status, length of hospital stay, anesthesia method, preoperative Harris score/self-care ability upon admission; postoperative rehabilitation exercise compliance; types of comorbidities. *p* < 0.05 indicated that there was a statistical difference.

## 3 Results

### 3.1 Baseline data evaluation

Compared with patients in the lower score group, patients in the higher score group exhibited higher levels of education, length of hospital stay, Harris score 30 days after discharge, satisfaction with discharge, and compliance with rehabilitation exercise, with statistically significant differences (*p* < 0.05) (Table 1).

**Table 1:** Comparison of baseline data between these two groups.

Item		Lower score group (189)	Higher score group (142)	*p*-value
Discharge readiness		86.27 ± 6.13	95.39 ± 3.55	<0.001
Age (year)		66 (60, 71)	66 (58, 75.75)	0.633
Gender (%)	Male	76 (40.2)	53 (37.3)	0.675
	Female	113 (59.8)	89 (62.7)	
BMI		23.72 ± 3.16	23.75 ± 3.35	0.937
Height (cm)		159.93 ± 7.59	160.68 ± 8.48	0.397
Weight (kg)		60.75 ± 9.87	61.18 ± 10.24	0.701
Education level (%)	Illiterate/Primary school	83 (43.9)	38 (26.8)	0.004
	Technical secondary school/Secondary school	80 (42.3)	84 (59.1)	
	College/University/Master’s degree	26 (13.8)	20 (14.1)	
Marital status (%)	Married	160 (84.7)	116 (81.7)	0.57
	Divorced/Widowed	29 (15.3)	26 (18.3)	
Length of hospital stay (day)		10 (8, 13)	11 (9, 15)	0.025
Anesthesia method (%)	General anesthesia	109 (57.7)	70 (49.3)	0.161
	Lumbar spinal cord/Spinal canal anesthesia	80 (42.3)	72 (50.7)	
Adverse events within 30 days after discharge (%)	Yes	2 (1)	3 (2.1)	0.426
Comorbidities (%)	Yes	119 (63)	106 (74.6)	0.033
Self-care ability	Admission	95 (70, 95)	92.5 (55, 95)	0.312
	Discharge	85 (80, 90)	85 (80, 90)	0.824
	30 days after discharge	95 (90, 100)	95 (90, 100)	0.525
Harris score	Admission	45 (32, 52)	46 (26, 50)	0.517
	Discharge	64 (61, 70)	65 (60, 71)	0.278
	30 days after discharge	77.32 ± 5.73	80.8 ± 7.21	<0.001
Discharge satisfaction		91.1 (89.3, 94.6)	92.9 (89.4, 96.35)	0.001
Rehabilitation exercise compliance	Postoperative	53 (48, 56)	56 (52, 58)	<0.001
	30 days after discharge	54 (49, 59)	58 (55, 60)	<0.001

### 3.2 Logistic regression analysis of the correlation between discharge readiness and Harris score or self-care ability

There was no significant difference in preoperative Harris score between the higher score group and the lower score group (*p* = 0.083). The multivariate regression results showed a correlation between the discharge readiness and changes in the Harris score 30 days after discharge (compared with that before surgery) of patients undergoing THA (*p* < 0.001). However, no sufficient evidence was observed to prove that discharge readiness was related to changes in Harris score at discharge, self-care ability at admission, self-care ability at discharge, and self-care ability 30 days after discharge (compared with that before surgery) (*p* > 0.05) (Table 2).

**Table 2:** Correlation between discharge readiness and Harris score or self-care ability.

	Coefficient	Standard deviation	*T* value	*p*-value
Preoperative Harris score	0.012	0.090	0.132	0.895
Harris score changes at discharge^*^	0.120	0.095	1.258	0.209
Harris score changes 30 days after discharge^*^	0.336	0.093	3.624	<0.001
Self-care ability at admission	−0.093	0.151	−0.616	0.538
Self-care ability changes at admission^**^	0.110	0.143	0.772	0.441
Self-care ability changes 30 days after discharge^**^	0.089	0.141	0.633	0.527

*It refers to the change in Harris score compared with that before surgery. **It refers to the change in self-care ability compared with that before surgery.

### 3.3 Mixed effects model analysis of the correlation between discharge readiness and Harris score or self-care ability over time

Patients undergoing THA had a 12.95-point higher Harris score 30 days after discharge than that at discharge (*p* < 0.001). The Harris score of the higher score group was on average 4.002 points higher than that of the lower score group (*p* = 0.004). From the evaluation at different time points after discharge, there was a significant difference in the Harris score between both groups (interaction term *p* < 0.001) (Table 3).

**Table 3:** Correlation between discharge readiness and Harris score over time.

	Coefficient	Standard deviation	*T* value	*p*-value
Time	12.952	0.531	24.390	<0.001
Discharge readiness grouping	4.002	1.394	2.871	0.004
Age	−0.015	0.041	−0.369	0.712
Gender	−0.111	0.889	−0.125	0.901
Height	0.051	0.063	0.807	0.420
Weight	−0.040	0.041	−0.957	0.339
Anesthesia method	−0.445	0.666	−0.669	0.504
Marital status	−0.080	0.908	−0.088	0.930
Length of hospital stay	−0.118	0.084	−1.399	0.163
Comorbidities	0.263	0.747	0.352	0.725
Postoperative rehabilitation exercise compliance	0.059	0.060	0.973	0.331
Preoperative Harris score	0.186	0.032	5.905	<0.001
Time: Discharge readiness	3.843	0.811	4.740	<0.001

Patients undergoing THA had a self-care ability score 30 days after discharge of 11.53 points higher compared with that at discharge (*p*  < 0.001). However, there was insufficient evidence to prove that there was a significant difference in the average self-care ability score between both groups (*p* = 0.888). From the evaluation at different time points after discharge, a significant difference in self-care ability score between both groups cannot be observed (interaction term *p* = 0.792) (Table 4).

**Table 4:** Correlation between discharge readiness and self-care ability over time.

	Coefficient	Standard deviation	*T* value	*p*-value
Time	11.534	0.579	19.910	0.001
Discharge readiness grouping	0.203	1.441	0.141	0.888
Age	−0.076	0.034	−2.206	0.028
Gender	−0.407	0.741	−0.549	0.583
Height	−0.097	0.053	−1.828	0.068
Weight	0.043	0.034	1.264	0.207
Anesthesia method	−0.365	0.548	−0.667	0.505
Marital status	2.038	0.738	2.761	0.006
Length of hospital stay	−0.063	0.071	−0.894	0.372
Comorbidities	−0.088	0.608	−0.145	0.885
Postoperative rehabilitation exercise compliance	−0.071	0.050	−1.426	0.155
Self-care ability changes at admission	0.131	0.015	8.439	0.001
Time: discharge readiness	0.233	0.884	0.264	0.792

## 4 Discussion

Total hip arthroplasty (THA) is suitable for patients with advanced hip joint diseases, severe functional impairment, and ineffective conservative treatment caused by various reasons (14). With the advancement of medicine, the ERAS concept proposed by Kehlet has been widely applied in orthopedic fields, such as THA, thus bringing good news to patients and challenges to medical staff (15). The discharge readiness of patients is closely related to the ERAS concept (16), and medical staff can evaluate the physical, mental, and social conditions of patients during hospitalization, thereby guiding their further recovery after discharge. However, in the field of orthopedics, especially THA, there are few reports on the relationship between the discharge readiness and functional indicators of patients. In view of this, a single-center retrospective cohort study was conducted based on the ERAS concept to explore the correlation between discharge readiness and Harris score or self-care ability in patients undergoing THA. These scientific efforts may provide new strategies and plans for orthopedic medical staff to achieve higher diagnosis, treatment, and nursing levels. This is also conducive to the postoperative physical and mental recovery of patients.

In this study, the information on 331 patients undergoing THA was retrospectively collected, and these patients were divided into the lower score group and the higher score group based on their discharge readiness scores. The baseline data (age, gender, height, weight, and BMI) were consistent between the two groups with no significant differences. However, patients with higher discharge readiness scores exhibited higher levels of education, length of stay, Harris score 30 days after discharge, satisfaction with discharge, and compliance with rehabilitation exercises. Kaya et al. (17) found that age, gender, marital status, educational status, the presence or absence of caregivers at home after discharge, and length of hospital stay were predictive factors for discharge readiness. Insufficient preparation for discharge increased the risk of 30-day unplanned readmission and 30-day death. Yang et al. (18) verified that the education at discharge and the discharge status of rehabilitation institutions were related to the readiness of colorectal cancer patients for discharge readiness. Medical staff should improve the quality of discharge guidance and pay more attention to patients who are transferred to rehabilitation institutions for further treatment after discharge. Similar to this study, Heine et al. (19) explored the influencing factors of discharge readiness in patients undergoing THA. They confirmed that “confidence,” “family and friends,” and “feeling safe” were important factors that affected the discharge readiness of these patients. These patients hoped to be safe in the hospital and at home, and their confidence and sense of security from friends and family at home were very important. Patients with a sense of security presented better preparation for discharge. Scatizzi et al. (20) found that age and cancer risks can affect the discharge preparation time of patients with colorectal cancer after surgery. Borgwardt et al. (21) made an exploration based on patients with unicompartmental knee arthroplasty in the field of orthopedics and found that adopting accelerated care procedures could shorten the hospitalization time of such patients, which also demonstrated the importance of discharge readiness under the ERAS concept. Based on existing studies, there were consistent baseline data for the basic situation of patients with high and low discharge readiness scores after THA. However, education level, length of hospital stay, Harris score 30 days after discharge, discharge satisfaction, and rehabilitation exercise compliance might be influencing factors on the discharge readiness of patients undergoing THA. Overall, the higher the education level of patients, the easier it was for patients to understand the medical advice of medical staff and follow the doctor’s instructions for preoperative preparation and postoperative exercise. This contributed to the recovery of hip joint functions. Therefore, patients with a higher education level regained better hip joint functions, and they were more likely to meet discharge standards. Besides, these patients presented higher satisfaction with diagnosis and treatment and more trust in medical staff, and they were more likely to cooperate with subsequent rehabilitation plans. Their confidence and trust in friends and family were also stronger. This also gave us a reminder that medical staff needed to be more patient with patients with low discharge readiness scores, communicate with them in plain language, help them correctly understand the disease, increase their compliance with medical advice, enhance their confidence in facing the disease, and improve their discharge readiness, which contributed to their recovery after discharge. For patients with high discharge readiness scores, medical staff could spend more time on developing more precise perioperative diagnosis and treatment plans. When communicating with these patients, they needed to improve their awareness and implement the collaborative diagnosis and treatment to transform the patients with low discharge readiness scores into those with high discharge readiness ones, which conduced to better recovery of these patients. Therefore, it is necessary to improve the discharge preparation of all patients under the guidance of the ERAS concept, which would be of great significance for the rehabilitation of patients undergoing THA.

In this study, it was also found that there was a correlation between discharge readiness and changes in the Harris score 30 days after discharge (compared with that before THA). The Harris score 30 days after discharge was higher than that at discharge. Patients with higher discharge readiness scores also had higher Harris scores compared with those with low discharge readiness scores. From the evaluation at different time points after discharge, there was a significant difference in the Harris score between the higher and lower score groups. However, there was no sufficient evidence to prove that discharge readiness was related to changes in the Harris score at discharge (compared with that before surgery). Söderman et al. (22) evaluated the effectiveness of the Harris score in THA. They compared the results with that of the Western Ontario and McMaster University Osteoarthritis Index and the Medical Outcomes Study 36-Item Short Form Health Survey. Eventually, they concluded that the Harris score could be applied to patients undergoing THA. Kladny et al. (23) demonstrated that the Harris score increased significantly in patients after hip arthroplasty, and it was not related to the gender, age, comorbidities, and weight of patients. Hersnase et al. (24) analyzed the Harris score and SF-36 score in the 5-year follow-up after THA with two different prostheses. They found that both scores were effective in relevant assessments. Weel et al. (25) evaluated the effectiveness of the Harris score in THA. They maintained that compared with the Oxford score, the Harris score presented higher evaluation effectiveness, while the Oxford score exhibited better internal consistency and reliability. From this, it could be observed that the Harris score was very important in the evaluation of THA. In this study, the Harris score generally increased after THA, and hence the Harris score at discharge and 30 days after discharge would be higher than that at admission. The higher the Harris score, the better the recovery of hip joint functions. In addition, these patients also exhibited higher satisfaction with diagnosis and treatment, and they presented more confidence in obtaining better exercise outcomes after discharge. Further, they also obtained higher discharge readiness scores, which constituted a virtuous cycle. As a result, there were significant differences in the Harris score at different time points after discharge between both groups of patients.

The results of this study also validated that the self-care ability of patients was significantly improved 30 days after discharge compared with that at discharge. There was no significant difference in the self-care ability at different time points after discharge between both groups. No sufficient evidence was observed to prove that discharge readiness was related to self-care ability at admission, self-care ability at discharge, and changes in self-care ability at 30 days after discharge (compared with that before surgery). This indicated that THA could affect the patient’s self-care ability, possibly due to the fact that the original symptoms of patients could be resolved within 30 days after discharge, which improved the subjective feeling of patients compared with that before surgery and at discharge, thereby improving their self-care ability. From the completion of the surgery to discharge, patients might experience certain stress reactions and postoperative discomfort, such as hip joint pain, due to the influence of surgery. This affected the subjective feeling and self-care ability of these patients, resulting in a lack of significant improvement in their self-care ability before and after discharge. The discharge readiness level of patients was only determined by their readiness to return to society, but their subjective perception or tolerance of hip joint symptoms would not change with the discharge readiness level. Only when it became apparent that hip joint symptoms were mitigated could their self-care ability be improved. This also explained that the discharge readiness level did not affect the self-care ability of these patients. In addition, the changes in time points would not affect the self-care ability of the two groups of patients. Konnyu et al. (26) conducted a systematic review of different rehabilitation plans after hip arthroplasty and found that different rehabilitation plans did not affect the self-care ability or quality of life of patients. Sakia et al. (27) also investigated hip surface arthroplasty, THA through the anterolateral approach, and THA through the posterolateral approach. They found that there was no significant difference in postoperative self-care ability among these three groups. Ren et al. (28) confirmed that early rehabilitation care could significantly improve the self-care ability of older adult patients undergoing hip arthroplasty. Su et al. (29) found that the comprehensive care for patients undergoing THA through the anterolateral approach could significantly improve their comfort level during treatment and their self-care ability after surgery. Based on domestic and foreign studies, it could be found that the self-care ability of patients undergoing THA contributed to the recovery of their hip joint functions. However, when patients did not receive relevant intervention on their self-care ability during the perioperative period, their self-care ability changes would not be affected. This could also provide indirect explanations for the relationship between the discharge readiness and the self-care ability of patients in this study. We believe that with a clear understanding of the relationship between discharge readiness and Harris score or self-care ability, and even other influencing factors in patients undergoing total hip replacement surgery, we could contribute to the rapid recovery program, also known as “Fast-Track” in patients undergoing THA (30). This will be beneficial for us to develop rehabilitation plans to similar patients, allowing them to be discharged as soon as possible and return to society.

Of course, there are certain limitations in this study. Firstly, this is a retrospective study, with a relatively low level of evidence in evidence-based medicine. Hence, it is necessary to design prospective studies to validate the results of this study. Secondly, it is a single-center study, which may lead to some bias in the research data. Therefore, it is required to conduct joint multicenter studies in different regions and institutions in the future.

In conclusion, there was a correlation between discharge readiness and changes in Harris score 30 days after discharge (compared with that before surgery) in THA patients. The Harris score and self-care ability 30 days after discharge were higher than those at the time of discharge. Patients in the higher score group exhibited a higher Harris score compared with those in the lower score group. From the evaluation at different time points after discharge, there was a significant difference in the Harris score between both groups. These results are expected to provide guidance for the physical and mental recovery of patients undergoing THA under the ERAS concept. Furthermore, these findings may contribute to higher diagnosis, treatment, and nursing levels of orthopedic medical staff.

## Data Availability

The raw data supporting the conclusions of this article will be made available by the authors, without undue reservation.

## References

[1] Bastian JD. Total hip arthroplasty-current challenges. Medicina (Kaunas). (2023) 59:1011. doi: 10.3390/medicina59061011 37374215 PMC10305566

[2] Ferguson RJ Palmer AJ Taylor A Porter ML Malchau H Glyn-Jones S. Hip replacement. Lancet. (2018) 392:1662–71. doi: 10.1016/S0140-6736(18)31777-X 30496081

[3] Kehlet H. Enhanced postoperative recovery: good from afar, but far from good? Anaesthesia. (2020) 75:e54–61. doi: 10.1111/anae.14860 31903577

[4] Kaye AD Urman RD Cornett EM Hart BM Chami A Gayle JA. Enhanced recovery pathways in orthopedic surgery. J Anaesthesiol Clin Pharmacol. (2019) 35:S35–9. doi: 10.4103/joacp.JOACP_35_18, PMID: 31142957 PMC6515716

[5] Frassanito L Vergari A Nestorini R Cerulli G Placella G Pace V. Enhanced recovery after surgery (ERAS) in hip and knee replacement surgery: description of a multidisciplinary program to improve management of the patients undergoing major orthopedic surgery. Musculoskelet Surg. (2020) 104:87–92. doi: 10.1007/s12306-019-00603-4, PMID: 31054080

[6] Fenwick AM. An interdisciplinary tool for assessing patients' readiness for discharge in the rehabilitation setting. J Adv Nurs. (1979) 4:9–21. doi: 10.1111/j.1365-2648.1979.tb02984.x, PMID: 254690

[7] Celio DA Poggi R Schmalzbauer M Rosso R Majno P Christoforidis D. ERAS, length of stay and private insurance: a retrospective study. Int J Color Dis. (2019) 34:1865–70. doi: 10.1007/s00384-019-03391-2, PMID: 31595311

[8] Kilpiö O Härkki PSM Mentula MJ Väänänen A Pakarinen PI. Recovery after enhanced versus conventional care laparoscopic hysterectomy performed in the afternoon: a randomized controlled trial. Int J Gynaecol Obstet. (2020) 151:392–8. doi: 10.1002/ijgo.13382, PMID: 32961589

[9] YaDeau JT Fields KG Kahn RL LaSala VR Ellis SJ Levine DS. Readiness for discharge after foot and ankle surgery using peripheral nerve blocks: a randomized controlled trial comparing spinal and general anesthesia as supplements to nerve blocks. Anesth Analg. (2018) 127:759–66. doi: 10.1213/ANE.0000000000003456, PMID: 29847387

[10] Sztain JF Machi AT Kormylo NJ Abramson WB Madison SJ Monahan AM. Continuous Adductor Canal versus continuous femoral nerve blocks: relative effects on discharge readiness following Unicompartment knee arthroplasty. Reg Anesth Pain Med. (2015) 40:559–67. doi: 10.1097/AAP.0000000000000279 26115189

[11] Ikutomo H Nagai K Tagomori K Miura N Okamura K Okuno T. Incidences and circumstances of falls among women following total hip arthroplasty on long-term follow-up. J Orthop Sci. (2023) 28:577–82. doi: 10.1016/j.jos.2021.12.013, PMID: 35063335

[12] Huang KY Chang CH Yu KC Hsu CH. Assessment of quality of life and activities of daily living among elderly patients with hypertension and impaired physical mobility in home health care by antihypertensive drugs plus acupuncture: a CONSORT-compliant, randomized controlled trial. Medicine (Baltimore). (2022) 101:e29077. doi: 10.1097/MD.0000000000029077 35356935 PMC10684176

[13] Liu P Zhao J Xu C Li Q. Mediating effects of psychological characteristics of mindfulness on family concern and rehabilitation compliance of patients undergoing hip arthroplasty. Chin J Mod Nurs. (2021) 27:316–22. doi: 10.3760/cma.j.cn115682-20200527-03588

[14] Zaballa E Dennison E Walker-Bone K. Function and employment after total hip replacement in older adults: a narrative review. Maturitas. (2023) 167:8–16. doi: 10.1016/j.maturitas.2022.09.005, PMID: 36302339

[15] Peng HM Tong BD Li Y Wang W Li WL Gao N. Mitigation of postoperative urinary retention among total joint replacement patients using the ERAS protocol and applying risk-stratified catheterization. ANZ J Surg. (2022) 92:2235–41. doi: 10.1111/ans.17847, PMID: 35716163

[16] Yang J He YH Jiang LL Zhou ZG Li K. Analysis of the status quo and influencing factors of short-term quality of life after discharge in colorectal cancer patients following enhanced recovery after surgery pathway. Zhonghua Yi Xue Za Zhi. (2019) 99:1707–11. doi: 10.3760/cma.j.issn.0376-2491.2019.22.005, PMID: 31216816

[17] Kaya S Sain Guven G Aydan S Kar A Teleş M Yıldız A. Patients' readiness for discharge: predictors and effects on unplanned readmissions, emergency department visits and death. J Nurs Manag. (2018) 26:707–16. doi: 10.1111/jonm.12605, PMID: 29573007

[18] Yang J He Y Jiang L Li K. Colorectal patients' readiness for hospital discharge following management of enhanced recovery after surgery pathway: a cross-sectional study from China. Medicine (Baltimore). (2020) 99:e19219. doi: 10.1097/MD.0000000000019219, PMID: 32080116 PMC7034666

[19] Heine J Koch S Goldie P. Patients' experiences of readiness for discharge following a total hip replacement. Aust J Physiother. (2004) 50:227–33. doi: 10.1016/S0004-9514(14)60112-4, PMID: 15574111

[20] Scatizzi M Feroci F Zironda A Lenzi E Baraghini M Garzi A. Enhanced recovery after surgery efficacy in an older patients and high-risk population affected by colorectal cancer: a more than 1000 patients experience. G Chir. (2019) 40:559–68. PMID: 32007121

[21] Borgwardt L Zerahn B Bliddal H Christiansen C Sylvest J Borgwardt A. Similar clinical outcome after unicompartmental knee arthroplasty using a conventional or accelerated care program: a randomized, controlled study of 40 patients. Acta Orthop. (2009) 80:334–7. doi: 10.3109/17453670903035559, PMID: 19513890 PMC2823215

[22] Söderman P Malchau H. Is the Harris hip score system useful to study the outcome of total hip replacement? Clin Orthop Relat Res. (2001) 384:189–97. doi: 10.1097/00003086-200103000-00022 11249165

[23] Kladny B Albrecht C Haase I Swoboda B. Inpatient rehabilitation of patients following total hip replacement--a study using the Harris hip score. Z Orthop Ihre Grenzgeb. (2001) 139:536–40. doi: 10.1055/s-2001-19237, PMID: 11753776

[24] Hersnaes PN Gromov K Otte KS Gebuhr PH Troelsen A. Harris hip score and SF-36 following metal-on-metal total hip arthroplasty and hip resurfacing – a randomized controlled trial with 5-years follow up including 75 patients. BMC Musculoskelet Disord. (2021) 22:781. doi: 10.1186/s12891-021-04671-1, PMID: 34511090 PMC8436430

[25] Weel H Lindeboom R Kuipers SE Vervest TMJS. Comparison between the Harris- and Oxford hip score to evaluate outcomes one-year after total hip arthroplasty. Acta Orthop Belg. (2017) 83:98–109. PMID: 29322902

[26] Konnyu KJ Pinto D Cao W Aaron RK Panagiotou OA Bhuma MR. Rehabilitation for Total hip arthroplasty: a systematic review. Am J Phys Med Rehabil. (2023) 102:11–8. doi: 10.1097/PHM.0000000000002007, PMID: 35302955 PMC9464790

[27] Sakai T Abe H Nakamura N Hamada H Takao M Sugano N. Differences in activities of daily living after hip arthroplasty among hip resurfacing, anterolateral THA, and posterolateral THA: a propensity score matched analysis. J Artif Organs. (2019) 22:84–90. doi: 10.1007/s10047-018-1069-7, PMID: 30251057

[28] Ren J Liu X. The effect of early rehabilitation nursing on daily life self-care ability of elderly patients undergoing hip replacement. Clin Res Pract. (2017) 2:186–7. doi: 10.19347/j.cnki.2096-1413.201726092

[29] Su Q. Nursing intervention on comfort and postoperative self-care ability of patients undergoing total hip arthroplasty through anterolateral approach. Chin J Woman Child Health Res. (2017) 28:184–5.

[30] Drosos GI Kougioumtzis IE Tottas S Ververidis A Chatzipapas C Tripsianis G. The results of a stepwise implementation of a fast-track program in total hip and knee replacement patients. J Orthop. (2020) 21:100–8. doi: 10.1016/j.jor.2020.03.004, PMID: 32255989 PMC7114635

